# Epidemiology of serotype 19A isolates from invasive pneumococcal disease in German children

**DOI:** 10.1186/1471-2334-13-70

**Published:** 2013-02-05

**Authors:** Mark van der Linden, Ralf René Reinert, Winfried V Kern, Matthias Imöhl

**Affiliations:** 1 Institute for Medical Microbiology, National Reference Center for Streptococci, Department of Medical Microbiology, University Hospital (RWTH), Pauwelsstr. 30, Aachen, 52074, Germany; 2 Division of Infectious Diseases, Department of Medicine, Albert-Ludwigs-University, Freiburg, Germany

**Keywords:** *Streptococcus pneumoniae*, Serotype 19A, Germany

## Abstract

**Background:**

This study presents an analysis of 159 serotype 19A isolates from IPD in children before and after the general recommendation for childhood pneumococcal conjugate vaccination in Germany in July 2006. Vaccination formulations used were PCV7, PCV10 (from April 2009) and PCV13 (from Dec. 2009, replacing PCV7).

**Methods:**

Isolates from invasive pneumococcal disease in children were serotyped using the Quellung reaction, tested for antibiotic susceptibility and analysed for their multi locus sequence type.

**Results:**

In an analysis of 3328 isolates from invasive pneumococcal disease (IPD) in children that were sent to the German National Reference Center for Streptococci between July 1997 and June 2011, we show that the proportion of 19A isolates ranged between 1.7 and 4.2% in the period 1997 to 2006. After the recommendation for pneumococcal conjugate childhood vaccination, which was issued in July 2006, the proportion of 19A isolates increased significantly to 15.0% in 2010/11. Eight clonal complexes (CC) and groups accounted for 77.2% and 65.3% of all serotype 19A isolates before and after vaccination, respectively. While three CCs and several STs were not detected after vaccine introduction, four CCs and several STs first appeared after vaccination, including three ST320 isolates that could be traced to recent imports from the US, UK and India. The proportion of penicillin-nonsusceptible and of multidrug-resistant 19A isolates moderately increased after vaccine introduction. A significant increase in the use of cephalosporins and azithromycin was noted post-vaccination (p=0.00001 and p=0.0013 respectively).

**Conclusions:**

The prevalence of serotype 19A in Germany has increased significantly between July 2007 and June 2011. Possible reasons for this are the introduction of pneumococcal conjugate vaccination, increased use of cephalosporins and azithromycin, import of multidrug-resistant isolates and increased reporting.

## Background

*Streptococcus pneumoniae* remains a major cause of infectious disease globally, especially in children. Invasive pneumococcal disease (IPD) causes over 1 million deaths among children worldwide [1]. The most important virulence factor of *S. pneumoniae* is the polysaccharide capsule. To date, 94 capsular types, or serotypes, have been described.

Since 2001, a pneumococcal conjugate vaccine (PCV7) has been available, covering the seven serotypes most prevalent in invasive pneumococcal disease (4, 6B, 9 V, 14, 18C, 19 F and 23 F). In the US, a strong reduction in the incidence of PCV7 vaccine serotype IPD has been observed after a national immunization program (NIP) with PCV7 began in 2000 [2]. This reduction was accompanied by an increase in IPD caused by non-PCV7 vaccine serotypes, however, it was to a much lower extent than the reduction. The most prevalent of the emerging non-PCV7 serotypes after vaccination in the US was serotype 19A, and currently serotype 19A is the most common serotype in IPD in children in the US [3] also suggesting that antibodies to the serotype 19F polysaccharide, included in PCV7, do not offer cross-protection against 19A infections.

It has been controversially discussed whether the increase in serotype 19A infection was a direct result of the vaccination program with PCV7 (‘serotype replacement’) and if the phenomenon could be contributed solely to vaccine use or whether other factors contributed to these changes in the serotype epidemiology. Even though the majority of US post-NIP 19A still belong to clonal complex (CC) 199, an increasing number of isolates belong to a single multidrug-resistant (i.e. resistant to more than 2 classes of antibiotics; MDR) clone, ST320 [4-6]. It was therefore speculated that, among several other factors, the increase could be a result of selective pressure due to the excessive use of antibiotics.

Globally, inconsistent observations were made, e.g., an increase of MDR clones of 19A has been observed among IPD in children in Israel and in South Korea, countries with very limited or no access to PCV7 at that time [7-9].

In Europe, pneumococcal vaccination programs for PCV7 have existed in most countries since 2006. Most of these countries report a considerable reduction of vaccine serotypes among IPD in children. However, many European countries also report an increase in non-vaccine serotype IPD, especially in serotype 19A [10-12].

The aim of the current study was to determine possible changes in the rate of reported infections and the clonal composition of serotype 19A IPD isolates before and after the widespread use of PCV7 in 2006 in Germany, and to evaluate possible factors driving these epidemiological changes, including PCV7 uptake and antibiotic usage.

## Methods

### Study material

Serotype 19A isolates were obtained from an ongoing surveillance study on IPD in German children under the age of 16 from July 1997 to June 2011 [13]. This passive laboratory surveillance system collects isolates from invasive pneumococcal disease in children under 16 years of age, from diagnostic laboratories all over Germany. Participation of laboratories is voluntary. A nationwide active prospective hospital surveillance of IPD in German pediatric hospitals is performed by the survey unit for rare pediatric diseases in Germany (Erhebungseinheit für Seltene Pädiatrische Erkrankungen in Deutschland, ESPED) [14] and, together with the laboratory surveillance system, allows for the calculation of IPD incidences among German children, using capture recapture between cases reported by hospitals and cases reported by diagnostic laboratories [15,16]. Calculated incidences indicate that for approximately half of the IPD cases, an isolate is sent to the German National Reference Center. *S. pneumoniae* isolated from blood, CSF or other normally-sterile body sites were included in the study. Since pneumococcal infections appear to cluster around the winter months, isolates are grouped according to ‘pneumococcal seasons’ (July in one year – June in the following year).

### Pneumococcal vaccination

A recommendation for pneumococcal conjugate vaccination for all children under 2 years of age was issued by the German standing committee on vaccination (STIKO) in July 2006. Germany does not have a national immunization plan, but costs of recommended vaccinations are reimbursed by health insurance companies. A seven-valent pneumococcal conjugate vaccine (PCV7, serotypes: 4, 6B, 9 V, 14, 18C, 19 F, 23 F) was licensed in Germany in 2001, followed by PCV10 (serotypes PCV7 + 1, 5, 7 F) in April 2009 and PCV13 (serotypes PCV10 + 3, 6A, 19A) in December 2009 (replacing PCV7). The choice of vaccine is up to the parents.

### Serotyping

Pneumococcal isolates were serotyped by Neufeld’s Quellung reaction using type and factor sera provided by the Statens Serum Institut, Copenhagen, Denmark.

### Susceptibility testing

All strains were tested for antibiotic minimal inhibitory concentrations (MIC) using the broth microdilution method as recommended by the CLSI [17]. The microtiter plates (Sensititre NLMMCS10, TREK Diagnostic Systems Ltd., East Grinstead, UK) contained penicillin G, clarithromycin, clindamycin and tetracycline with cation adjusted Mueller-Hinton broth (Oxoid, Wesel, Germany) and 5% lysed horse blood. The current CLSI criteria were applied for interpretation [18]. To assess the development of penicillin resistance and in the definition of multidrug resistance, the ‘oral’ penicillin breakpoints were used (≤0.06 μg/ml, 0.12-1 μg/ml, ≥2 μg/ml), since they give better insight into resistance development over time. Otherwise, the parenteral breakpoints (meningitis: ≤0.06 μg/ml, - , ≥0.12 μg/ml; non-meningitis: ≤2 μg/ml, 4 μg/ml, ≥8 μg/ml) were used. Breakpoints used for other antibiotics were: clarithromycin, clindamycin: ≤0.25 μg/ml, 0.5 μg/ml, ≥1 μg/ml and tetracycline: ≤2 μg/ml, 4 μg/ml, ≥8 μg/ml. Isolates were considered multidrug-resistant when they were resistant to more than two different classes of antibiotics.

### Multilocus sequence typing

Multilocus sequence typing of selected pneumococcal isolates was performed as described previously [19]. Briefly, internal fragments of the *aroE, gdh, gki, recP, spi, xpt,* and *ddl* genes were amplified by PCR from chromosomal DNA with the described primer pairs. A special allelic profile is provided by the alleles at each of the seven loci and their sequence type (ST) is defined. The allelic profiles were compared with each other and with other isolates in the pneumococcal MLST database using software available at http://www.mlst.net. Clusters of related STs were grouped into clonal complexes (CCs) using the program eBURST on the global database on http://www.mlst.net.

### Antibiotic and vaccine use

Data on vaccination rates are not publicly available in Germany. Sales data for PCV7 and PCV13 were obtained from Pfizer Pharma GmbH, Berlin, Germany. Yearly antibiotic sales data from the German health insurance (Gesetzliche Krankenversicherung, GKV) database (dispensed drugs based on analysis of all prescriptions) were obtained from the scientific institute of the public health insurances in Germany (Wissenschaftliches Institut der AOK (Allgemeine Ortskrankenkassen), WidO), Berlin, a central institute which summarizes national drug prescription data for compulsory health insurances (90% of the German population). The data were expressed in defined daily doses (DDD) per 1,000 persons covered by the insurance and day, using the Anatomical Therapeutic Chemical (ATC) classification methodology and current DDD definitions (http://www.whocc.no) – analogous to the DDD per 1,000 inhabitants and day format used in the European Surveillance of Antibiotic Consumption (ESAC) project (http://www.esac.ua.ac.be).

### Statistical methods

Differences in proportions were tested by Fisher’s exact test with a two-sided P value of <0.05 considered significant. Differences in distributions were tested with a two-sample t-test with P values of <0.05 considered significant. Analyses were conducted using the Analysis Toolpak from MS Excel and GraphPad (http://graphpad.com/quickcalcs/contingency2).

### Ethical statement

An ethical approval was not required since the study did not involve human subjects, material or data.

## Results

From July 1997 until June 2011, a total of 3328 isolates from invasive pneumococcal disease were sent to the German National Reference Center for Streptococci. Isolate numbers varied from a minimum of 152 in 1998/99 to a maximum of 295 in 2006/07 (Table 1). The average number of isolates per year was 238. Isolates were sent in from all over Germany, with no geographical bias. A total of 159 infections were caused by serotype 19A. The percentage of serotype 19A isolates remained below 5% until 2006, and then increased constantly over time, with a significantly higher proportion (8.1%) during the post-PCV-vaccination period when compared to the pre-PCV-vaccination period (2.8%) (Table 1). Interestingly, the stepwise introduction of higher-valent vaccines in 2009 only slightly reduced the increase in serotype 19A.

**Table 1:** Isolates from IPD in children from Germany from July 1997 to June 2011, proportion of serotype 19A and antibiotic resistance among serotype 19A isolates

	**Number of isolates per season**	**Number of serotype**	Number of 19A isolates resistant* (%)				
**Season**		**19A (%)**	**PEN I+R**	**CLA R**	**CLI R**	**TET R**	**MDR**
Pre-PCV vaccination period							
1997-1998	167	7 (4.2)	1 (14.3)	1 (14.3)	0 (0.0)	1 (14.3)	0 (0.0)
1998-1999	152	6 (3.9)	2 (33.3)	1 (16.7)	1 (16.7)	1 (16.7)	1 (16.7)
1999-2000	190	6 (3.2)	1 (16.7)	2 (33.3)	2 (33.3)	3 (50.0)	2 (33.3)
2000-2001	238	5 (2.1)	0 (0.0)	1 (20.0)	1 (20.0)	1 (20.0)	1 (20.0)
2001-2002	241	7 (2.9)	3 (42.9)	1 (14.3)	1 (14.3)	2 (28.6)	1 (14.3)
2002-2003	239	4 (1.7)	0 (0.0)	1 (25.0)	1 (25.0)	0 (0.0)	1 (25.0)
2003-2004	272	6 (2.2)	2 (33.3)	1 (16.7)	1 (16.7)	1 (16.7)	0 (0.0)
2004-2005	286	11 (3.8)	5 (45.5)	4 (36.4)	3 (27.3)	5 (45.5)	2 (18.2)
2005-2006	291	6 (2.1)	2 (33.3)	2 (33.3)	1 (16.7)	4 (66.7)	1 (16.7)
Total	2076	58 (2.8^#^)	16 (27.6^†^)	14 (24.1)	11 (19.0)	18 (31.0)	9 (15.5)
Post-PCV vaccination period (PCV7)							
2006-2007	295	12 (4.1)	3 (25.0)	5 (41.7)	5 (41.7)	6 (50.0)	5 (41.7)
2007-2008	241	11 (4.6)	4 (36.4)	2 (18.2)	1 (9.1)	4 (36.4)	1 (9.1)
2008-2009	249	19 (7.6)	12 (63.2)	5 (26.3)	4 (21.1)	8 (42.1)	5 (26.3)
Post-PCV vaccination period (PCV10, PCV7/13)							
2009-2010	233	24 (10.3)	11 (45.8)	7 (29.2)	7 (29.2)	6 (25.0)	7 (29.2)
2010-2011	234	35 (15.0)	8 (22.9)	8 (22.9)	8 (22.9)	7 (20.0)	8 (22.9)
Total (post PCV)	1252	101 (9.9^#^)	38 (37.6^†^)	27 (26.7)	25 (24.8)	31 (30.7)	26 (25.7)

^#^The Fisher’s exact two-tailed P values are ≤0.001.

^†^The two-sampled t-test exact two-tailed P values are ≤0.05.

*PEN penicillin; *CLA* clarithromycin; *CLI* clindamycin; *TET* tetracycline; *R* resistant, I intermediately resistant'; *MDR* multidrug-resistant.

Forty-four 19A isolates (27.7%) were from patients with meningitis. Among these, 15 isolates (34.1%) were resistant to penicillin, according to the new (parenteral) CLSI breakpoints (MIC ≥0.12 μg/ml) [17]. Penicillin resistance rates post-vaccination were considerably higher (40.0%) than pre-vaccination (26.3%), though the difference was not statistically significant (p=0.52). Among the 115 non-meningitis isolates, only intermediate penicillin G resistance (MIC=4 μg/ml) was found (4 cases, 3.5%).

As described in the materials and methods section, the ‘oral’ penicillin breakpoints (which correspond to the old CLSI breakpoints [20]), were used to assess the development of penicillin non-susceptibility among the 19A isolates, since they give better insight into resistance development over time. Applying these breakpoints, 54 isolates (9 resistant, 45 intermediately resistant) were classified as penicillin non-susceptible (NS), and the proportion of penicillin non-susceptible isolates increased significantly (p=0.002) between the pre- and the post-PCV-vaccination period (Table 1). Rates of resistance to macrolides, clindamycin, and tetracycline were relatively high, but did not change significantly over time (Table 1). Seventy-five isolates were resistant to at least one, 59 to at least two and 35 to three or more classes of antibiotics (using oral penicillin breakpoints). The latter isolates were classified as multidrug-resistant (MDR) (Table 1). The proportion of isolates with resistance to at least one drug and the proportion of MDR isolates also increased moderately between the pre- and post-PCV-vaccination period, but the differences were statistically not significant (≥1 drug: p=0.7418, MDR: p=0.1655).

**Figure 1:**
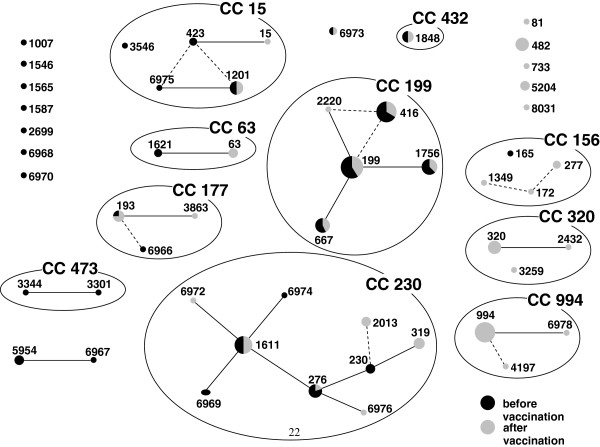
**eBURST snapshot of 19A isolates from IPD in children in Germany before (black) and after (gray) the introduction of pneumococcal conjugate vaccination.** Circle size is proportional to the amount of isolates.

Multilocus sequence typing was performed for 158 of the 159 isolates included in this study (one isolate could not be regrown). The most common sequence types found were ST199 (10.7%), ST416 (9.4%) and ST994 (8.8%) (Figure 1). When categorized according to clonal complex (CC) CC199 was the most prevalent (30.4%), followed by CC230 with 18.4%.

When the clonal composition of the serotype 19A isolates before and after the start of the pneumococcal conjugate vaccine recommendation in July 2006 was compared, several changes in clonal composition could be observed. Eight CCs or groups accounted for 77.2% and 65.3% of all isolates before and after vaccination, respectively. Three CCs and 7 other STs were not detected after vaccine introduction. Clonal complexes 81, 320, 733 and 994 and STs 482, 5204 and 8031 were detected only after vaccine introduction. A significant prevalence increase after the introduction of vaccination was only detected for CC994 (p=0.0006) and CC320 (p= 0.0496) (Table 2).

**Table 2:** ST distribution among serotype 19A isolates from IPD in German children, before and after introduction of pneumococcal conjugate vaccine

**CC**	**Before vaccination**			**After vaccination**			
	**STs**	**n=**	**%**	**STs**	**n=**	**%**	**p-value**
473	3301, **3344**	2	3.5		0	0	0.1287
2699	2699	1	1.8		0	0	0.3608
3111	**6968**	1	1.8		0	0	0.3608
group of two (5480)	1565	1	1.8		0	0	0.3608
group of two (5954, 6967)	5954(3), 6967	4	7.0		0	0	**0.0158**
group of two (8025)	6970	1	1.8		0	0	0.3608
singleton	1587	1	1.8		0	0	0.3608
singleton	1546	1	1.8		0	0	0.3608
singleton	1007	1	1.8		0	0.0	0.3608
15	423(2), 1201(3), **3546**, 6975	7	12.3	1201(3), **15**	4	4.0	0.0582
63	**1621**(**1**+1)	2	3.5	**63(3)**	3	3.0	1.0000
156	165	1	1.8	172, 277(2), 1349	4	4.0	0.6545
177	**193**, 6966	2	3.5	**193(3), 3863**	4	4.0	1.0000
199	199(7), 416(5), 667(3), 1756(3)	18	31.6	199(10), **416**(**3**+7), 667(4), 1756(5), 2220	30	29.7	0.5899
230	**230**(**3**), 276, 1611(5), 6969, 6974	11	19.3	**276**(**2**+2), **319**(**4**), 1611(5), 2013(3), 6972, 6976	18	17.8	0.8329
432	1848(2)	2	3.5	**1848(2)**	2	2.0	0.6199
group of two (2668)	6973	1	1.8	6973	1	1.0	0.5358
81			0	**81**	1	1.0	1.0000
320			0	**320**(**5**), **2432**, 3259	7	6.9	**0.0496**
733			0	733	1	1.0	1.0000
994			0	994(14), 4197, 6978	16	15.8	**0.0006**
group of two (2653)			0	482(6)	6	5.9	0.0880
singleton			0	5204(3)	3	3.0	0.5537
singleton			0	8031	1	1.0	1.0000
**total**		**57**	**100**		**101**	**100**	

MDR isolates are indicated in bold. P-values from Fisher’s exact test, bold numbers are considered statistically significant (p<0.05).

The increased proportion of MDR isolates was mainly associated with increased proportions of CC230 and CC320. Of note, in the period before vaccination, only three MDR isolates with intermediate penicillin resistance were found, all three belonging to CC230 (ST230). After vaccination, 9 MDR isolates with high resistance to penicillin were found, belonging to CC230 (ST276, n=2), CC320 (ST320, n=5, ST2432, n=1) and CC81 (ST81, n=1) (Table 3). Three of the ST320 isolates could be traced back to children that had recently come to Germany. On the clinical data sheets sent in with the isolates, it was stated that one child had entered from the US shortly before being infected, one from the UK, and a third had recently spent a longer holiday in India. In fact these were the first, second and third ST320, serotype 19A isolates ever detected in Germany (Nov. 2008, Mar. 2009 and Dec. 2009, respectively). The only earlier detected ST320 isolate was a serotype 19F, found in June 2006. The other two ST320 serotype 19A isolates (Jan. and May 2010), as well as the ST2432 isolate (Apr. 2009), had no data which indicated a foreign origin.

**Table 3:** MDR serotype 19A isolates from IPD in German children, before and after introduction of pneumococcal conjugate vaccine

**Date of Isolation**	**Diagnosis**	**Source**	**CC**	**ST**	PEN* (μg/ml)	CLA* (μg/ml)	CLI* (μg/ml)	TET* (μg/ml)
**Pre-vaccination**								
01.03.1999		CSF	230	230	0.25	32	32	32
09.09.1999		Blood	473	3344	0.03	32	32	32
27.01.2000		Blood	3111	6968	0.015	4	2	32
28.11.2000		Blood	63	1621	0.015	4	2	16
31.12.2001		Blood	230	230	0.12	32	32	16
01.01.2003		Blood	n.d.	n.d.	0.015	32	32	2
04.01.2005		Swab mastoid	230	230	0.25	32	32	32
07.01.2005		Blood	177	193	0.015	32	32	32
23.11.2005	Sepsis	Blood	15	3546	0.015	32	32	32
**Post-vaccination**								
29.09.2006	others	Blood	432	1848	0.015	16	16	16
25.11.2006	Otitis media	Blood	199	416	0.015	16	16	16
21.12.2006	Pneumonia	Blood	230	276	2	16	16	16
08.01.2007	Meningitis	CSF	177	193	0.015	16	16	16
08.06.2007	Pneumonia	Pleuraempyema	177	193	0.015	16	16	16
20.10.2007	Sepsis	Blood	177	193	0.015	16	16	16
05.11.2008	Pneumonia	Blood	320	320	4	16	16	16
29.03.2009	Sepsis	Blood	320	320	4	16	16	16
20.04.2009	Pleuritis	Drainage thorax	320	2432	4	16	16	16
10.05.2009		Blood	81	81	2	8	0.5	16
29.06.2009		Blood	230	319	0.25	16	16	16
06.10.2009	Mastoiditis	Swab mastoid	230	319	0.5	16	16	16
07.11.2009	Sepsis	Blood	199	416	0.015	16	16	16
11.12.2009	Sepsis	Blood	320	320	2	16	16	0.25
15.01.2010	Pneumonia	Pleural fluid	320	320	2	16	16	16
23.02.2010		Blood	63	63	0.25	16	16	16
03.04.2010	Pneumonia	Blood	432	1848	0.015	16	16	4
05.05.2010	Sepsis	Blood	320	320	4	16	16	16
08.07.2010	Otitis media	Blood	15	15	0.5	16	16	0.5
20.07.2010		CSF	63	63	0.12	16	16	16
16.09.2010	Phlegmon	Blood	230	319	0.5	16	16	0.5
26.09.2010	Pneumonia	Blood	230	276	2	16	4	16
28.12.2010		Blood	199	416	0.015	16	16	16
30.03.2011		Blood	63	63	0.06	16	16	16
13.04.2011	Meningitis	CSF	177	3863	0.015	16	16	16
30.06.2011	Meningitis	CSF	230	319	1	16	16	0.5

*PEN, penicillin; *CLA,* clarithromycin; *CLI*, clindamycin; *TET*, tetracycline.

**Figure 2:**
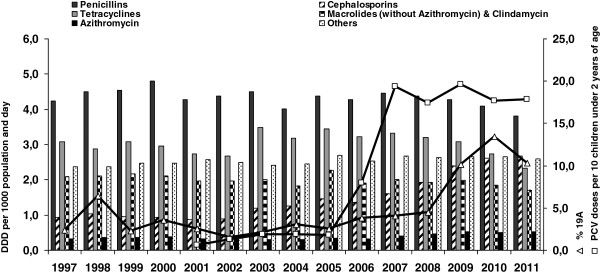
Yearly antibiotic prescription rates (DDD per 1,000 population and day, left axis), marketed doses of PCV per 10 children under 2 years of age (squares, right axis), and percentage of IPD cases caused by serotype 19A in Germany (triangles, right axis).

Data on antibiotic use in Germany were available for 1997–2011. Significant increases in cephalosporin and azithromycin use were noted when comparing the pre- and post-vaccination periods (p=0.00001 and p=0.0013 respectively). However, with 23.4% of all macrolide use in 2011, the proportional use of azithromycin remains relatively low in Germany. Penicillin, macrolides (without azithromycin) + clindamycin, and tetracycline use increased incrementally, but did not show significant change when comparing the pre- and post- vaccination periods (Figure 2).

The number of marketed doses of PCV7 (since 2001) and PCV13 (since Dec. 2009) in Germany was around 200,000 per year before the general vaccination recommendation. After the recommendation was issued in July 2006, this amount rose to an average of 2 million doses per year (Figure 2). The birth cohorts in Germany have been an average 736,000 births/year from 1997–2006 and an average 675,000 births/year from 2007–2011.

## Discussion

The general recommendation for childhood pneumococcal conjugate vaccination was issued in Germany in July 2006, and full reimbursement of vaccination started in January 2007. The uptake of the vaccine was estimated to be almost 80% in 2007 [15] and is probably higher to date. We were interested in evaluating the effects of vaccine introduction on the prevalence and clonal composition of serotype 19A among isolates from IPD in German children <16 years of age.

An important limitation of our study was that the referral of isolates to the reference center was incomplete since reporting IPD is not mandatory in Germany. However, reported cases show no regional bias, and referral patterns have been constant for years. Using our capture-recapture incidence calculations, we determined that before the vaccination recommendation, a sample had been sent to the National Reference Center for 40-50% of all IPD cases. This percentage increased to 50-60% after vaccination introduction [21]. Furthermore, overall numbers of reported isolates have fallen since the start of vaccination in 2006. Moreover, it should be noted that these numbers include all cases in children under 16 years of age, of which many are still unvaccinated. The number of cases in children under 2 years of age has fallen more sharply: 2005–2006: 154, 2010–2011: 100.

The proportion of 19A isolates in Germany increased significantly after vaccine introduction. However, an increase in the incidence of infection due to serotype 19A (as calculated using capture-recapture) was observed only in 2008/2009 [22], but not in 2007/2008 [15]. Therefore, the increased proportion of 19A isolates in the first two seasons following the vaccination recommendation could have been due to increased reporting [21]. Additionally, the increasing use of cephalosporins and perhaps of azithromycin in the general population may have been a factor in the change in 19A epidemiology, including the increased proportion of resistant 19A isolates. The increase in serotype 19A was only slightly affected by the introduction of higher-valent vaccines in Germany. A reason for this could be that PCV13 was only introduced in December 2009, and it is too early to see more clear effects in the 2010–2011 pneumococcal season. However, when the 19A prevalence data are grouped by calendar year (Figure 2) an indication for a decrease is observed.

In the US, an increase in 19A was reported among Alaskan children [23], as well as in the general childhood population [4]. Richter *et al.* reported most of the penicillin-resistant and multidrug-resistant isolates after vaccination to have serotype 19A [24]. Spain and France also report increases in 19A after the start of vaccination programs [10,12]. However, reports from South Korea, Belgium and Israel show an increase in serotype 19A isolates from IPD in children before the introduction of pneumococcal conjugate vaccination [7,8,11]. Therefore, the increase in 19A is unlikely to be caused only by vaccination.

Despite the increases in cephalosporin and azithromycin use reported here, antibiotic prescription rates in Germany are generally low. Therefore, the selective advantage in carriage for resistant isolates, like MDR 19A, will be lower. This might be the reason why these clones do not (yet) persist in Germany. The only MDR 19A isolates reported before introduction of the vaccine belonged to CC230 (ST230), a more common clone in Germany, which also persists post-vaccination (ST319, ST276). However, these isolates show a considerably lower MIC for penicillin. MDR 19A ST320 isolates have also been reported from Spain and Italy, and MDR 19F ST320 isolates from Poland (http://spneumoniae.mlst.net/).

All of the observed STs in this study have been correlated to serotype 19A or 19F before. (http://www.mlst.net). Moore *et al.*, Beall *et al.* and Pillai *et al.* have described ST320 as the most prevalent multi-drug-resistant serotype 19A clone in the US and in Canada, respectively [4,6,25]. Of the newly-appeared STs after vaccination start in Germany, seven were MDR, of which six belonged to CC320. Interestingly, three of these isolates (all ST320) were from children that had recently come to Germany from abroad. Moreover, these were the first three ST320, serotype 19A isolates ever detected among children with IPD in Germany. This shows that at least some of the MDR 19A isolates that appeared after introduction of the vaccine in Germany are recent imports from countries where antibiotic pressure is higher than in Germany.

Still, a majority of the post-vaccination serotype 19A isolates are drug-susceptible and belong to the same clonal complexes as pre-vaccination 19A isolates. This shows that the main burden of serotype 19A is caused by the expansion of existing clones, although the fact that CCs 320 and 994 were detected significantly more often after vaccination introduction could indicate that a shift in the population of serotype 19A pneumococci in Germany is at hand. In a study on pneumococcal carriage in children in Massachusetts, Hanage *et al*. found that the most common serotype 19A clonal complex before and after vaccination was CC199 and that the observed increase was due to ST320 and ST695 [26]. Relatedly, in a recent paper from Portugal, Aguiar *et al.* show that the increase in 19A after the start of vaccination is due to an expansion of existing lineages (CC230) that are MDR [27]. They did not find any CC320 isolates.

After the issue of the general recommendation for pneumococcal vaccination in July 2006, the amount of sold doses of PCV7 rose strongly from 200,000 to 2 million per year (Figure 2). The increased use of the vaccine will have reduced carriage of 7-valent vaccine serotypes among children, thereby creating a niche for other serotypes. A study in the Netherlands showed increased carriage of serotype 19A in vaccinated children as compared with unvaccinated controls [28].

Our analysis shows that, after the start of pneumococcal conjugate vaccination in Germany, the epidemiology of serotype 19A in invasive pneumococcal disease among children has changed. Even though the majority of the clones has remained the same, several STs were not detected after the start of vaccination, while other STs were only detected after vaccination had started. Among these newly-detected STs are a number of MDR isolates, with high penicillin resistance.

## Conclusion

The increase in serotype 19A IPD isolates seems to be related to several factors. Increased awareness may have led to a higher amount of reported isolates. The extended use of PCV7 has potentially created a niche in carriage, which may have been occupied by 19A. The three first MDR 19A (ST320) isolates detected among children with IPD in Germany were shown to be imported from countries with a relatively high antibiotic pressure. Increasing use of cephalosporins and azithromycin might have favored the selection and/or expansion of penicillin-non-susceptible 19A clones.

## Competing interests

ML has been a member of advisory boards for and has received research grants and speakers honorary fees from Pfizer, GSK, Merck and SanofiPasteurMSD. RRR is currently an employee of Pfizer pharmaceuticals. MI and WK report no competing interests.

## Authors’ contributions

WK provided the data on antibiotic usage, performed the statistical analysis and helped to draft the manuscript. MI, RRR and ML participated in the laboratory analyses. RRR and ML conceived the study. ML drafted the manuscript. All authors read and approved the final manuscript.

## Pre-publication history

The pre-publication history for this paper can be accessed here:

http://www.biomedcentral.com/1471-2334/13/70/prepub
